# Regulation of gap junction intercellular communication by connexin ubiquitination: physiological and pathophysiological implications

**DOI:** 10.1007/s00018-019-03285-0

**Published:** 2019-09-09

**Authors:** Max Zachrisson Totland, Nikoline Lander Rasmussen, Lars Mørland Knudsen, Edward Leithe

**Affiliations:** 1 Department of Molecular Oncology, Institute for Cancer Research, Oslo University Hospital, 0424 Oslo, Norway; 2 K.G. Jebsen Colorectal Cancer Research Centre, Oslo University Hospital, Oslo, Norway; 3 Present Address: Department of Medical Biology, University of Tromsø, Tromsø, Norway

**Keywords:** Cataract, Cancer, Electrical synapse, Heart arrhythmia, Heart ischemia, Lens, NEDD4, Ubiquitin

## Abstract

Gap junctions consist of arrays of intercellular channels that enable adjacent cells to communicate both electrically and metabolically. Gap junctions have a wide diversity of physiological functions, playing critical roles in both excitable and non-excitable tissues. Gap junction channels are formed by integral membrane proteins called connexins. Inherited or acquired alterations in connexins are associated with numerous diseases, including heart failure, neuropathologies, deafness, skin disorders, cataracts and cancer. Gap junctions are highly dynamic structures and by modulating the turnover rate of connexins, cells can rapidly alter the number of gap junction channels at the plasma membrane in response to extracellular or intracellular cues. Increasing evidence suggests that ubiquitination has important roles in the regulation of endoplasmic reticulum-associated degradation of connexins as well as in the modulation of gap junction endocytosis and post-endocytic sorting of connexins to lysosomes. In recent years, researchers have also started to provide insights into the physiological roles of connexin ubiquitination in specific tissue types. This review provides an overview of the advances made in understanding the roles of connexin ubiquitination in the regulation of gap junction intercellular communication and discusses the emerging physiological and pathophysiological implications of these processes.

## Introduction

Gap junctions are plasma membrane domains containing arrays of intercellular channels that allow for the direct transfer of ions and small molecules (< ~ 1.2 kDa) between cells [1]. In vertebrates, gap junction channels are formed by a family of transmembrane proteins called connexins, which in humans constitutes 20 members [2]. Connexins are expressed in almost all cell types of the human body and have essential roles during development as well as in the adult organism. In providing a pathway for electrical communication, gap junctions are fundamental to the function of excitable cells, such as neurons, cardiomyocytes and smooth muscle cells [3]. Moreover, by enabling the intercellular exchange of small metabolites and second messengers, gap junctions have a plethora of essential roles in non-excitable tissues, including the regulation of cell proliferation and differentiation and the maintenance of tissue homeostasis [4, 5]. There is also increasing evidence that connexins have important cell physiological functions that go beyond their ability to form canonical gap junctions. For instance, recent studies have demonstrated that connexins are involved in cell–cell communication via tunneling nanotubes and extracellular vesicles [6]. Moreover, connexin-based channels at the plasma membrane that are not assembled into gap junctions, also known as hemichannels, have central roles in autocrine and paracrine signaling by providing a pathway for the exchange of ions and small molecules between the intracellular and extracellular milieus [7]. In addition, the intracellular domains of connexins can interact with other proteins, including components of the cytoskeleton, such as tubulin, and cell signaling pathways, such as cyclin E and β-catenin [8–11]. Through such protein–protein interactions, connexins can modulate cell growth, differentiation, migration and other cellular processes in channel-independent manners. The multifaceted roles of connexins in human physiology are reflected by the fact that inherited or acquired alterations in connexins are associated with numerous diseases, including heart failure, neuropathologies, deafness, skin disorders, cataracts and cancer [12–14]. Obtaining a better understanding of the molecular basis underlying the loss of connexin function in disease pathogenesis may have important therapeutic implications [15–17].

The connexin pool that constitutes gap junctions is continuously replaced due to the formation of new intercellular channels at their edges and the removal of old channels from their center by endocytosis and subsequent degradation in lysosomes [18–22]. This dynamic nature of gap junctions is reflected by the observation that the connexins have a high turnover rate in most tissue types, typically displaying half-lives of 1.5-5 h [23–25]. Substantial evidence suggests that cells can modulate the connexin degradation rate in response to various extracellular or intracellular cues to alter the number of functional gap junction channels at the plasma membrane [20, 26–32].

An increasing body of experimental work suggests that post-translational modification of connexins by ubiquitination has an important role in regulating the level of gap junction intercellular communication. Ubiquitination has been suggested to be involved in controlling the degradation of de novo synthesized connexins at the endoplasmic reticulum, in modulating the rate of gap junction endocytosis, and in sorting connexins to lysosomes via either the autophagosomal or the endolysosomal pathways. In recent years, researchers have also started to provide insights into the physiological roles of connexin ubiquitination in specific tissue types and how dysregulation of this process may contribute to loss of gap junctions during disease pathogenesis. This review provides an up-to-date overview of the current understanding of the role of connexin ubiquitination in the regulation of gap junctional intercellular communication. We also discuss the emerging physiological and pathophysiological implications of connexin ubiquitination, focusing on the heart, lens and central nervous system. The implications of connexin ubiquitination for cancer pathogenesis have previously been reviewed elsewhere [33] and this topic will, therefore, not be covered here.

## Connexin biosynthesis and assembly into gap junctions

**Fig. 1:**
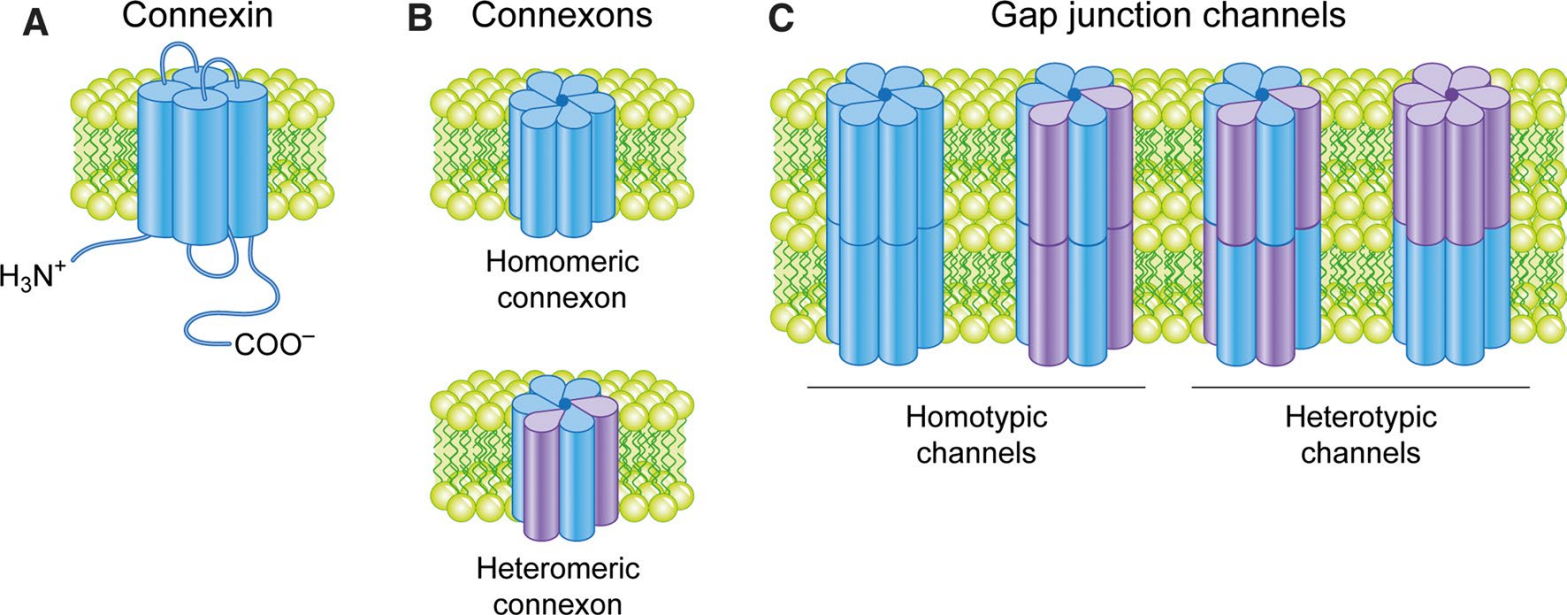
Connexins, connexons and gap junction channels. **a** Connexins have four transmembrane domains, which are connected by two extracellular loops and one intracellular loop. The N- and C-termini are both located in the cytosol. **b** Connexins form hexamers called connexons. Connexins can combine with either the same or different connexin isoforms, forming homomeric or heteromeric connexons, respectively. **c** Connexons form gap junction channels by interacting with either identical homomeric or heteromeric connexons in adjacent cells, forming homotypic channels, or with different homomeric or heteromeric connexons, forming heterotypic channels

Connexin proteins are named according to their approximate molecular weight (in kDa) [1]. The best studied and among the most ubiquitously expressed connexin isoforms in human tissues is connexin 43 (Cx43) [1]. The connexins are four-pass transmembrane proteins displaying their N- and C-terminal tails in the cytosol (Fig. 1a) [34]. The transmembrane domains and the two extracellular loops are highly conserved between the connexin family members. In contrast, the intracellular loop and N- and C-terminal tails exhibit great variation in their length and amino acid sequence between connexins. These regions have important roles in modulating gap junction channel gating and intracellular trafficking of connexins. For instance, the C-terminal tail of Cx43 contains multiple sites for protein–protein interactions and post-translational modifications that play important roles in the regulation of gap junctions [10, 35].

**Fig. 2:**
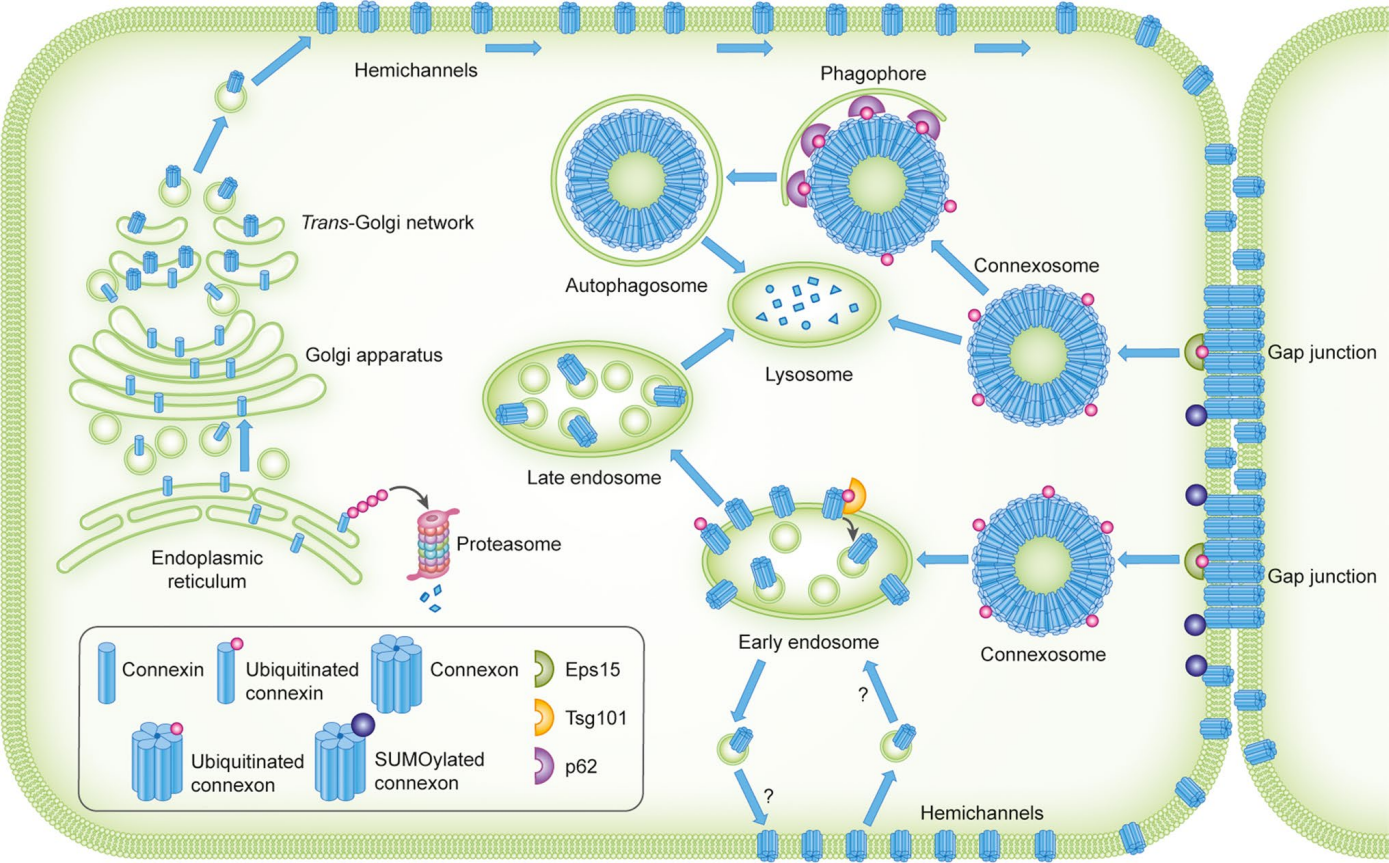
Intracellular trafficking and ubiquitination and SUMOylation of connexins. Connexins are co-translationally inserted into the endoplasmic reticulum and transported through the Golgi, through the *trans*-Golgi and to the plasma membrane. Along their trafficking to the plasma membrane, connexins oligomerize into connexons. A subpool of the newly synthesized connexins undergoes ERAD, in which connexins are retrotranslocated to the cytosol and degraded by proteasomes. In the plasma membrane, connexons can function as hemichannels or diffuse to the periphery of gap junctions, where they can dock with connexons in adjacent cells to form intercellular channels. During endocytosis of gap junctions, both membranes of the junctions are internalized into one of the cells, thereby forming a connexosome, also called an annular gap junction. Three different pathways for trafficking of connexins to lysosomes are illustrated in the figure: (1) direct fusion between connexosomes and lysosomes, (2) sequestration of the connexosome by a phagophore and subsequent fusion between an autophagosome containing the connexosome and a lysosome, and (3) transformation of the connexosome into a connexin-enriched, multivesicular endosome with a single limiting membrane, which is associated with the fusion between the connexosome and early endosomes. Connexins are then sorted from early endosomes via late endosomes to lysosomes. Connexons at the plasma membrane that are not assembled into gap junctions can also undergo endocytosis, but their intracellular trafficking is poorly characterized. They are possibly transported to early endosomes (indicated by a question mark). Following endocytosis, connexins may undergo recycling to the plasma membrane, possibly from the early endosomes (indicated by a question mark). Connexin ubiquitination has been suggested to be involved in ERAD of certain connexin isoforms, gap junction endocytosis, autophagy-mediated degradation of connexins and sorting of connexins from early endosomes to lysosomes. Eps15 has been suggested to bind to ubiquitinated Cx43 at the plasma membrane and to control gap junction endocytosis and autophagy-mediated degradation. Tsg101 is part of ESCRT and has been suggested to bind to ubiquitinated Cx43 at the limiting membrane of early endosomes and to regulate its sorting into the lumen of the endosome. p62 has been suggested to bind to ubiquitinated Cx43 after gap junction endocytosis and to be involved in the sequestration of connexosomes by autophagosomes. Cx43 has also been shown to be modified by SUMOylation, which has been suggested to stabilize Cx43 and consequently cause increased gap junction size

Connexins are co-translationally inserted into the endoplasmic reticulum [36, 37]. A subpool of newly synthesized connexins undergoes endoplasmic reticulum-associated degradation (ERAD), a process in which they are retrotranslocated into the cytosol and degraded by the proteasome (Fig. 2) [38–40]. Connexins that are spared from ERAD are transported from the endoplasmic reticulum via the Golgi apparatus and the *trans*-Golgi network to the plasma membrane [41, 42]. Along the secretory pathway from the endoplasmic reticulum to the plasma membrane, connexins oligomerize into hexameric structures called connexons [43–45]. Connexons can consist of either six similar connexin isoforms or a combination of different connexin isoforms, referred to as homomeric and heteromeric connexons, respectively (Fig. 1b) [46, 47].

In the *trans*-Golgi network, connexons are packaged into vesicles that are transported along microtubules to the plasma membrane [18, 21, 22, 48]. Connexons can be delivered to regions of un-apposed plasma membrane where they diffuse laterally in the lipid bilayer until they reach the periphery of existing gap junctions [18, 21, 49, 50]. Alternatively, connexons can be sorted directly to, or in the immediate vicinity of, existing gap junctions [22]. The connexons then dock with connexons in the plasma membrane of neighboring cells to form gap junction intercellular channels. Connexons can form channels either with identical homomeric or heteromeric connexons in adjacent cells (homotypic channels) or with channels with different homomeric or heteromeric connexons (heterotypic channels) (Fig. 1c) [46, 47]. The connexin composition of the gap junction channels strongly affects the conductance and selectivity of the channels.

In addition to full-length Cx43, six N-terminally truncated Cx43 isoforms can form as a result of internal translation initiation [51–54]. Emerging evidence indicates that such truncated versions of Cx43 have important roles in controlling the intracellular trafficking of full-length Cx43 [41, 42]. For instance, a truncated 20-kDa Cx43 isoform, termed GJA1-20 k, has been shown to increase the delivery of Cx43 hemichannels to cardiac intercalated discs to increase the gap junction plaque size [55]. In addition, truncated Cx43 isoforms may have important functions in the nucleus. In accordance with this notion, GJA1-20 k was recently demonstrated to control neural crest cell migration in vivo by directly regulating N-cadherin transcription [56].

## Endocytosis and degradation of gap junctions

During gap junction endocytosis, both membranes of the junction are internalized into one of the adjacent cells, thereby forming a double-membrane vesicle called an annular gap junction or connexosome [57, 58]. Either an entire gap junction or only a fragment of it can be internalized. The endocytosis of gap junctions involves clathrin [59–62]. Cx43 contains two functional tyrosine-based motifs (^265^YAYF^268^ and ^286^YKLV^289^) in its C-terminal tail that conform to the consensus YXXϕ (where Y is tyrosine, X is any amino acid and ϕ is an amino acid with a bulky hydrophobic side chain) [63, 64]. These motifs bind to the clathrin adaptor AP-2, which then recruits clathrin directly or indirectly via the clathrin adaptor Dab2 [63]. Other proteins shown to be involved in gap junction endocytosis include dynamin, 14-3-3 and the retrograde actin motor myosin-VI [62, 65–67].

Subsequent to gap junction endocytosis, connexins can follow different post-endocytic pathways before they are degraded in lysosomes (Fig. 2) [68–70]. Sometimes, connexosomes can fuse directly with lysosomes [30, 71–73]. In other situations, they can be sequestered by autophagosomes, which subsequently fuse with lysosomes [74–78]. Moreover, connexosomes have been suggested to be able to undergo a transformation into a connexin-enriched multivesicular endosome, which is associated with their fusion with early endosomes [79–82]. Some studies also indicate that following endocytosis, connexins can under certain conditions undergo recycling from endocytic compartments back to the plasma membrane [32, 75, 81, 83–85]. It has also been suggested that Cx43 can be transported directly from early secretory compartments to lysosomes without prior transport to the plasma membrane [30].

## Post-translational modifications of connexins

Connexins are subjected to several different types of post-translational modifications, which contribute to the regulation of gap junctions by different mechanisms [86]. Among the post-translational modifications of connexins that have been identified are phosphorylation, ubiquitination, SUMOylation, acetylation, methylation, hydroxylation and nitrosylation [86].

Kinases known to catalyze the phosphorylation of Cx43 include mitogen-activated protein kinase (MAPK), protein kinase C (PKC), casein kinase 1, protein kinase A, c-Src and v-Src [87]. Phosphorylation regulates several stages of the connexin “life cycle”. For instance, phosphorylation of Cx43 on serine or tyrosine residues located in the C-terminal tail has been shown to regulate Cx43 trafficking from the Golgi apparatus to the plasma membrane, the oligomerization of Cx43 into connexons, the assembly of connexons into gap junctions, the gating of gap junction channels, and gap junction endocytosis and degradation [88]. As described below, increasing evidence suggests that gap junction endocytosis and connexin degradation are controlled by complex crosstalk between connexin phosphorylation and ubiquitination.

## The ubiquitin system

Ubiquitin is a small globular protein that can be covalently attached to the ε-amino group of an internal lysine residue or free N-terminal α-amino group of the substrate, in a process known as ubiquitination [89]. A substrate can be subjected to different types of ubiquitin modifications, each of which can have different effects on the substrate. Proteins can be modified by a single ubiquitin, in a process known as monoubiquitination; by single ubiquitin moieties on several lysines, known as multiple monoubiquitination; or by a polyubiquitin chain, known as polyubiquitination. Ubiquitin has seven lysine residues that can all be involved in polyubiquitination, forming linear or branched ubiquitin chains. The distinct ubiquitin modifications are recognized by different effector proteins with specific ubiquitin-binding domains, thereby coupling the ubiquitination of a substrate to a downstream event, such as protein degradation, sorting to a specific subcellular location or assembly of a signaling complex [89]. For instance, modification of transmembrane proteins by polyubiquitin chains linked via Lys63 may act as a signal for their regulated endocytosis and subsequent targeting to lysosomes [89]. In contrast, modification of proteins by polyubiquitin chains linked via Lys48 may target proteins for degradation by the 26S proteasome [89].

Conjugation of ubiquitin to the target protein proceeds through a cascade mechanism that involves three enzymes: E1 ubiquitin-activating enzyme, E2 ubiquitin-conjugating enzyme and E3 ubiquitin ligase [90]. First, ubiquitin forms a high-energy thioester bond with a cysteine residue of an E1 ubiquitin-activating enzyme, a process requiring ATP. Ubiquitin is then transferred to an E2 ubiquitin-conjugating enzyme, forming a similar thioester linkage with a specific catalytic cysteine residue. E3 ubiquitin ligases recruit E2 ubiquitin-conjugating enzymes loaded with ubiquitin. The E3 ubiquitin ligase then recognizes a specific substrate and facilitates or directly transfers and conjugates ubiquitin to the target protein. E3 ubiquitin ligases can either conjugate ubiquitin to lysine residues in the target protein or conjugate ubiquitin to already attached ubiquitins, forming polyubiquitin chains [90].

The key regulatory determinants in protein degradation are the E3 ubiquitin ligases. While the human genome encodes two E1 activating enzymes and 37 E2 conjugating enzymes, it encodes over 600 E3 ubiquitin ligases [90]. E3 ubiquitin ligases are generally grouped into three subfamilies: the really interesting new gene (RING) type E3s, the U-box E3s and the homologous to E6-AP carboxyl terminus (HECT) domain-containing E3s [90].

Deubiquitinating enzymes are proteases that cleave ubiquitin off the substrate protein or within ubiquitin moieties in a polyubiquitin chain [91]. The human genome encodes approximately 100 deubiquitinating enzymes, the majority of which are cysteine proteases. The E3 ubiquitin ligases and deubiquitinating enzymes are key regulators of numerous intracellular processes and have been linked to the pathogenesis of a number of human diseases, including cancer and neurodegenerative disorders [92].

## Connexin ubiquitination

The first experimental evidence to indicate that Cx43 is ubiquitinated was provided by Laing and Beyer [27]. Using E36 Chinese hamster ovary cells containing a temperature-sensitive defect in the ubiquitin-activating protein E1, the authors also demonstrated that efficient Cx43 degradation requires an intact ubiquitin conjugation system [27]. In accordance with the notion that ubiquitination is involved in the regulation of gap junctions, Rütz and Hülser demonstrated by electron microscopy that ubiquitin is present in gap junction plaques, as determined by immunogold labeling of freeze-fractured plasma membranes [93]. Subsequent studies carried out in our laboratory indicated that endocytosis of gap junctions and Cx43 degradation may be regulated by crosstalk between Cx43 phosphorylation and ubiquitination. Using as a model system IAR20 rat liver epithelial cells, which express Cx43 endogenously, we found Cx43 ubiquitination to be induced by epidermal growth factor (EGF) [28]. The EGF-induced ubiquitination of Cx43 was MAPK dependent and was associated with increased endocytosis and degradation of gap junctions. Subsequently, treatment of cells with the tumor promoter 12-*O*-tetradecanoylphorbol-13-acetate (TPA), a potent activator of PKC, was found to result in strongly increased Cx43 ubiquitination, which correlated with enhanced gap junction endocytosis and degradation of Cx43 in lysosomes [94]. As determined by sodium dodecyl sulfate polyacrylamide gel electrophoresis, Cx43 in IAR20 cells appeared to be modified by one to four ubiquitin moieties [28, 94]. Analyses of the solubility of the ubiquitinated Cx43 pool in the detergent Triton X-100 indicated that Cx43 can be ubiquitinated while assembled into gap junctions and that Cx43 can remain ubiquitinated during gap junction endocytosis and along its sorting to early endosomes [81, 95].

Studies by Cuervo and colleagues have demonstrated that ubiquitination of Cx43 is induced in response to activation of macroautophagy by serum starvation [74]. Cx43 ubiquitination acts as a signal for gap junction endocytosis and autophagy-mediated degradation by recruiting the ubiquitin-binding protein Eps15 (epidermal growth factor receptor substrate 15) [74, 96] (Fig. 2). It has been suggested that Eps15 acts as an intermediate bridge molecule between ubiquitinated Cx43 and the autophagy machinery at the plasma membrane [74, 96]. Cx43 has also been shown to bind to the ubiquitin-binding autophagic adaptor p62 (also known as SQSTM1) [74, 76, 78]. The interaction between Cx43 and p62 increases in response to serum starvation or when a ubiquitin molecule is fused in-frame to the C-terminus of Cx43 to mimic Cx43 ubiquitination [74]. Cx43 co-localizes with p62 in intracellular vesicular compartments [74, 76, 78]. Moreover, depletion of p62 has been shown to result in accumulation of connexosomes [76]. These observations raise the possibility that ubiquitination of Cx43 may have a role in the sequestration of connexosomes by autophagosomes by recruiting p62 [74, 76, 78].

Cx43 ubiquitination has also been proposed to control its post-endocytic sorting from early endosomes to lysosomes [81]. Ubiquitinated forms of Cx43 have been suggested to be recognized by ubiquitin-binding proteins of the endosomal sorting complex required for transport (ESCRT), which is located at the limiting membrane of endosomes (Fig. 2). ESCRT is then thought to mediate the deubiquitination and subsequent sorting of Cx43 into the lumen of the endosomes [81]. In accordance with this notion, Cx43 has been shown to interact with the ubiquitin-binding protein Tsg101 (tumor susceptibility gene 101 protein), a member of ESCRT [97]. Depletion of Tsg101 results in increased Cx43 protein levels and enhanced levels of functional gap junctions at the plasma membrane, possibly due to increased recycling of Cx43 from early endosomes to the plasma membrane [81]. Moreover, under conditions in which Tsg101 is co-depleted with another ubiquitin-binding protein and member of ESCRT, Hrs (hepatocyte growth factor-regulated tyrosine kinase substrate), Cx43 accumulates at the limiting membrane of early endosomes in a hyperubiquitinated form [81].

In addition to Cx43, several other connexin isoforms have been shown to be ubiquitinated (Table 1). As well as playing a role in modulating gap junction endocytosis and post-endocytic sorting of connexins to lysosomes, connexin ubiquitination has been shown to be involved in ERAD. However, the involvement of ubiquitination in ERAD appears to be connexin isoform specific. For instance, ERAD of Cx43 occurs independently of ubiquitination, whereas ERAD of Cx32 and Cx40 involves polyubiquitination [98–100]. Kelly et al. have shown that ERAD of connexins is inhibited by mild forms of cytosolic stress at a step before their dislocation into the cytosol [40]. To investigate the mechanisms underlying this inhibition of connexin degradation, a mutant version of Cx32 associated with Charcot–Marie–Tooth X-linked peripheral neuropathy was studied [99]. This mutant Cx32 is confined to the endoplasmic reticulum and thus not subject to lysosomal degradation, and it was found to be modified by polyubiquitination [99]. Moreover, its level of polyubiquitination was found to be reduced in response to cytosolic stress, which was associated with its reduced ERAD [99]. These observations suggest that the inhibition of ERAD of connexins induced by cytosolic stress may be due to reduced connexin polyubiquitination.

**Table 1:** Overview of connexin isoforms reported to undergo ubiquitination

Connexin	Proposed role of ubiquitination	Cellular model system	References
Cx26	Proteasomal degradation of Cx26	HEK293T human embryo kidney cells exogenously expressing FLAG-Cx26 and HA-ubiquitin	[108]
Cx32	ERAD of Cx32	HeLa cervical cancer cells exogenously expressing Cx32-HKKSL	[100]
	ERAD of Cx32	CHO cells exogenously expressing Cx32-E208K and HA-ubiquitin	[99]
	Cx32 degradation	N2A murine neuroblastoma cells exogenously expressing Cx32-Myc	[103]
Cx36	Gap junction endocytosis and lysosomal degradation of Cx36	N2A exogenously expressing Cx36-eCFP and HA-ubiquitin	[125]
Cx40	ERAD of Cx40	HeLa cells exogenously expressing Cx40-wt or Cx40-G38D	[98]
Cx43	Proteasomal degradation of Cx43	E36 CHO cells	[27]
	Gap junction endocytosis and sorting of Cx43 from early endosomes to lysosomes	IAR20 rat liver epithelial cells	[28, 79, 81, 94]
	Gap junction endocytosis	HeLa cells exogenously expressing Cx43	[96]
	Gap junction endocytosis and autophagy-mediated degradation of Cx43	COS-7 monkey kidney fibroblasts exogenously expressing Cx43	[74, 162]
	Gap junction endocytosis and Cx43 degradation	C6 rat glioma cells exogenously expressing Cx43	[104]
	Gap junction endocytosis and Cx43 degradation	MDCK canine kidney cells exogenously expressing Cx43	[102]
	Gap junction endocytosis and sorting of Cx43 from early to late endosomes	HeLa cells exogenously expressing Cx43	[105]
	Proteasomal or lysosomal degradation of Cx43	MCF7 and BT474 breast cancer cells	[163]
	Autophagy-mediated degradation of Cx43	HEK293T cells exogenously expressing HA-Cx43	[107]
	Lysosomal degradation of Cx43	HEK293T cells exogenously expressing Cx43	[124]
	Gap junction endocytosis	Rat primary neonatal ventricular cardiomyocytes	[132]
	Gap junction endocytosis and lysosomal degradation of Cx43	Rat primary neonatal ventricular cardiomyocytes	[164]
	Remodeling of gap junctions during cardiac ischemia	HL-1 atrial cardiomyocyte cells	[133]
	Remodeling of gap junctions during cardiac ischemia	Rat heart Langendorff perfusion model (in vivo Cx43 ubiquitination)	[106]
	Gap junction endocytosis	HaCaT human keratinocytes exogenously expressing Cx43-wt or Cx43-S373A	[67]
	Lysosomal degradation of Cx43	Cultured neonatal rat ventricular cardiomyocytes	[138]
	Proteasomal degradation of Cx43	NN1003A rabbit lens epithelial cells	[146]
	Proteasomal degradation of Cx43	Lens (in vivo Cx43 ubiquitination)	[147]
	Proteasomal degradation of Cx43	Human lens epithelial cells	[147]
	Proteasomal degradation of Cx43	Neonatal rat spinal cord astrocytes	[165]
	Proteasomal degradation of Cx43	Primary rat astrocytes	[166]
Cx45.6	Proteasomal degradation of Cx45.6	N2A cells exogenously expressing eGFP-Cx45.6 and ubiquitin-Myc	[109]

*eCFP* enhanced cyan fluorescence protein, *CHO* Chinese hamster ovary, *eGFP* enhanced green fluorescent protein, *ERAD* endoplasmic reticulum-associated degradation, *MDCK* Madin–Darby Canine kidney, *N2A* neuro2A

An important question is which lysines in Cx43 and other connexin isoforms act as ubiquitination sites, and how ubiquitination of specific lysine residues affects gap junction levels. A study by Dunn et al. found that a mutant version of Cx43 with all lysines converted to arginines behaves similarly to Cx43-wt in the presence of proteasomal and lysosomal inhibitors [101]. The authors also demonstrated that proteasomal inhibition causes increased activation of Akt (protein kinase B), which is associated with enhanced Akt-mediated phosphorylation of Cx43 and, consequently, stabilization of Cx43 gap junctions [101]. The authors concluded that ubiquitination and proteasomal degradation of Akt, but not Cx43 ubiquitination, is involved in the regulation of Cx43 turnover. A subsequent study by Kells-Andrews, Margraf et al. indicated that two lysine residues located in the C-terminal tail of Cx43, Lys264 and Lys303 act as ubiquitin conjugation sites [102]. Mutation of these lysine residues to arginines resulted in the accumulation of Cx43 at the plasma membrane and increased its half-life. Under these conditions, Cx43 was found to be in a hyper-phosphorylated state, which could indicate that Cx43 ubiquitination is triggered by Cx43 phosphorylation under basal conditions. On the basis of these observations, the authors concluded that Cx43 ubiquitination is required for the constitutive gap junction internalization and degradation [102]. A recent study by Alaei et al. demonstrated that mutating five lysine residues in the C-terminal tail of Cx32 to arginines resulted in both reduced Cx32 acetylation and ubiquitination, and both these modifications were suggested to be involved in regulating Cx32 turnover [103]. Acetylation and ubiquitination were suggested to result in Cx32 stabilization and degradation, respectively [103]. The authors also demonstrated that mutating the five above-mentioned lysine residues to glutamine, which mimics acetylation, results in a stronger reduction in Cx32 ubiquitination as compared with when they are mutated to arginines [103]. These observations raise the possibility that Cx32 ubiquitination is negatively regulated by Cx32 acetylation, possibly through direct competition for the same lysine residues.

The available experimental evidence indicates that connexins can be subjected to different types of ubiquitin conjugation, including monoubiquitination (e.g., [104, 105]), multiple monoubiquitination (e.g., [94, 96]) and Lys63- or Lys48-linked polyubiquitination (e.g., [98–100, 102, 106–109]). It is likely that the type of ubiquitination that connexins are subjected to varies according to their subcellular localization. In future studies, it will be important to provide a more detailed understanding of the type of ubiquitin modifications that connexins are subjected to at different subcellular localizations and to better understand the functional consequences of the various types of modifications.

**Fig. 3:**
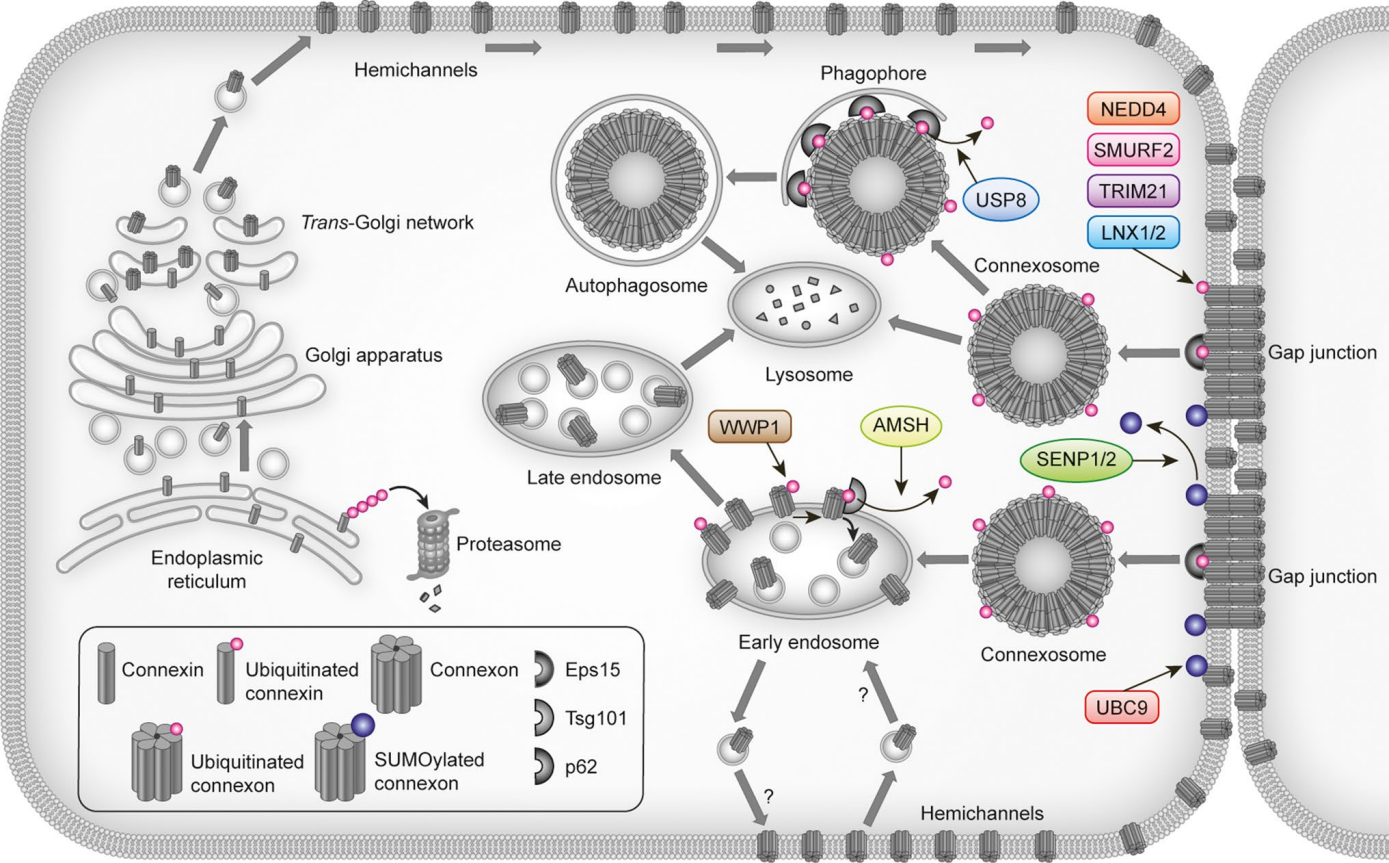
Overview of proteins involved in controlling gap junctions by connexin ubiquitination or SUMOylation. The figure depicts the proteins identified to date that participate in the regulation of gap junction intercellular communication through connexin ubiquitination or SUMOylation and the possible subcellular localizations where they may interact with connexins. For details, see Table 2 and the main text

In addition to ubiquitination, Cx43 has been found to be modified by the ubiquitin-like protein SUMO (small ubiquitin-related modifier) [110]. In contrast to ubiquitination, SUMOylation of Cx43 has been proposed to stabilize Cx43 at the plasma membrane (Figs. 2 and 3; Table 2) [110]. This raises the possibility that gap junction endocytosis and degradation are controlled by an interplay between Cx43 ubiquitination and SUMOylation.

**Table 2:** Overview of proteins reported to be involved in controlling gap junctions through connexin ubiquitination or SUMOylation

Protein type	Protein name	Connexin isoform	References
E3 ubiquitin ligase	NEDD4	Cx43	[74, 96, 105, 114, 115]
	SMURF2	Cx43	[79, 84]
	WWP1	Cx43	[124]
	TRIM21	Cx43	[104]
	LNX1 and -2	Cx36	[125]
Deubiquitinating enzyme	AMSH	Cx43	[126]
	USP8	Cx43	[107]
SUMO E2 conjugating enzyme	UBC9	Cx43	[110]
DeSUMOylating enzyme	SENP1/2	Cx43	[110]

*SENP1/2* SUMO/sentrin-specific peptidase, *UBC9* SUMO-conjugating enzyme UBC9

## Regulation of Cx43 gap junctions by NEDD4

The available data suggest that Cx43 is regulated by several E3 ubiquitin ligases (Fig. 3; Table 2). Among these, NEDD4 (neural precursor cell expressed developmentally down-regulated protein 4) is the best characterized. NEDD4 was originally discovered as a developmentally down-regulated gene in mouse brain [111]. NEDD4 is the founding member of the NEDD4 family of E3 ubiquitin ligases, which consists of nine members in humans [112]. The members of this protein family are characterized by a C-terminal catalytic HECT domain, a central protein–protein interaction region composed of 2–4 WW domains and an N-terminal membrane-targeting C2 domain. WW domains are protein modules that mediate protein–protein interactions by recognizing and binding proline-rich motifs and phosphorylated serine/threonine–proline sites in substrate proteins [113]. They are found in a variety of structural and signaling proteins and are involved in numerous cellular processes, including protein trafficking. WW domains consist of 38–40 amino acids and are characterized by two highly conserved tryptophan residues separated by 20–23 amino acids [113]. Based on their binding motif preference, WW domains are divided into five classes [113]. The WW domains of NEDD4 belong to class I, which bind to PY motifs (PPXY, where P is proline, X is any amino acid, and Y is tyrosine) in the substrate proteins. Cx43 contains a PY motif in its C-terminal tail (^283^PPGY^286^) [64].

In 2006, Leykauf et al. identified NEDD4 as the first E3 ubiquitin ligase to interact with Cx43. As determined by pull-down assays, rat NEDD4 (rNEDD4) was found to bind to the C-terminal tail of rat Cx43 (rCx43) and the interaction was shown to involve all three WW domains (WW1–3) of rNEDD4 [114]. Only WW2 was suggested to bind to the PY motif of Cx43, whereas the WW1 and WW3 domains were suggested to interact with other regions in the Cx43 C-terminal tail [114]. More recently, however, using various biophysical techniques, including nuclear magnetic resonance spectroscopy, Spagnol et al. provided evidence that all three WW domains of rNEDD4 bind to the PY motif of rCx43 [115]. WW2 was shown to have the highest binding affinity for the PY motif of rCx43, followed by WW3 and WW1. Mutation of the tyrosine in the PY motif (Y286A) led to a significant reduction in binding affinity for all WW domains, underscoring the importance of this residue in the interaction between the C-terminal tail of Cx43 and NEDD4.

Both the two aforementioned studies further indicated that phosphorylation of Cx43 is important for the interaction with NEDD4. Leykauf et al. found that treating cells with EGF, which causes MAPK-mediated phosphorylation of Cx43 at serine residues 255, 262, 279 and 282, increased the binding capacity of the WW3 domain of NEDD4 to Cx43 [114]. Interestingly, Ser279 and Ser282 are located just N-terminally to the PY motif, and phosphorylation of these residues (^279^pSPMpSPPGY^286^ where pS is phosphoserine) creates a potential class IV WW domain-binding motif [(pS/pT)P] adjacent to the PY motif [113]. Using peptides containing the PPXY motif, as well as the potential class IV motif, Spagnol et al. showed that phosphorylation of Ser282 significantly increased the affinity of all three WW domains of NEDD4 [115]. The binding affinity of WW2 was further increased when both Ser279 and Ser282 were phosphorylated, while phosphorylation of Ser279 alone had no influence. The contacts formed by phosphorylation of Ser279 and Ser282 were found to cause the region of the C-terminal tail of Cx43 encompassing the PY motif to form a horseshoe-shaped arrangement that fits into a groove formed by the NEDD4 WW2 domain. Taken together, these data raise the possibility that MAPK-mediated phosphorylation of the C-terminal tail of Cx43 increases the binding to NEDD4 in vivo, providing a possible mechanism for how the crosstalk between phosphorylation and ubiquitination of Cx43 is mediated.

The fact that the members of the NEDD4 family harbor multiple WW domains raises the possibility that tandem WW domain repeats may cooperate to enhance specificity and binding affinity [116]. For instance, the binding of the NEDD4 family member NEDD4-2 to its target SMAD2/3 first involves the phosphorylation-dependent binding of its WW3 domain to a site downstream of a PY motif, which positions the WW2 domain so that it can bind the PY motif, ultimately enhancing the total binding affinity [117]. The WW2 and WW3 domains of rNEDD4 have close proximity, raising the possibility that they could bind to the C-terminal tail of Cx43 in a cooperative manner. However, Spagnol et al. did not observe any cooperative binding of these two WW domains to a peptide containing the (pS/pT)P and PPXY motifs of Cx43, suggesting that they bind to Cx43 individually [115].

In accordance with the notion that NEDD4 binds to Cx43 via the PY motif, mutation of the first proline in this motif to a leucine (P283L) has been shown to result in reduced binding of NEDD4 to Cx43 as determined by co-immunoprecipitation [96], reduced Cx43 ubiquitination [96] and increased Cx43 protein levels [64]. Moreover, ectopic overexpression of NEDD4 in HeLa cells stably transfected with Cx43 or in C33A cervical carcinoma cells, which express Cx43 endogenously, has been shown to promote Cx43 ubiquitination and endocytosis, which is associated with a nearly complete loss of gap junctions [105]. The NEDD4-induced Cx43 endocytosis was found to be accompanied with loss of Cx43 protein levels due to its increased degradation via the endolysosomal pathway [105]. NEDD4 that contains an inactivating mutation in the catalytic cysteine of the HECT domain did not affect the Cx43 ubiquitination status, gap junction levels or the Cx43 protein level [105]. Thus, the ability of NEDD4 to promote ubiquitination, endocytosis and lysosomal degradation of Cx43 requires a functional HECT domain. NEDD4 is also involved in mediating the endolysosomal degradation of Cx43 in response to PKC activation [105]. In addition, NEDD4-induced ubiquitination of Cx43 has been suggested to act as a signal for gap junction endocytosis and autophagy-mediated degradation through recruitment of Eps15 [74, 96].

## Other E3 ubiquitin ligases involved in regulating Cx43 gap junctions

### SMURF2

SMURF1 and -2 (SMAD ubiquitination regulatory factor-1 and -2) are members of the NEDD4 family and were originally identified as important regulators of the transforming growth factor-β (TGF-β)/bone morphogenic protein signaling pathway by catalyzing the ubiquitination and degradation of SMAD proteins and the TGF-β receptor [118, 119]. Subsequent studies identified several other substrate proteins of SMURF1 and -2, and they were shown to play central roles in processes such as cell proliferation, differentiation, migration and senescence [120]. SMURF2 has been found to bind to Cx43, as determined by co-immunoprecipitation, and depletion of SMURF2 results in enhanced Cx43 levels and enlarged gap junctions, which is associated with increased gap junction intercellular communication [79]. SMURF2 also mediates the PKC-induced endocytosis and degradation of Cx43 [79]. In IAR20 cells, but not in HeLa cells, SMURF2 is also required for the remodeling of Cx43 gap junctions during mitosis [84]. However, it is unclear whether SMURF2 regulates gap junction endocytosis directly by catalyzing Cx43 ubiquitination or indirectly via another, yet unknown, protein [79].

### TRIM21

TRIM21 (cytosolic Fc receptor tripartite motif 21) is a member of the tripartite motif-containing (TRIM) protein family of RING E3 ubiquitin ligases [121]. TRIM21 links Fc-mediated antibody recognition to the ubiquitin proteasome system and has also been linked to the initiation of autophagy [121]. In addition, TRIM21 has been shown to regulate cell proliferation and cell death, and among its known substrate proteins is the cyclin-dependent kinase inhibitor p27 [122, 123]. Chen and colleagues have demonstrated that Cx43 interacts with TRIM21, as determined by both liquid chromatography tandem mass spectrometry and co-immunoprecipitation [104]. Through confocal microscopy, TRIM21 was found to co-localize with Cx43 at gap junctions. Analysis of TRIM21-mediated Cx43 ubiquitination in vitro with cell lysates of C6 rat glioma cells that exogenously expressed Cx43 (C6-Cx43) or astrocytes showed that mono- and diubiquitinated forms of Cx43 accumulated approximately 30 min after the initiation of the procedure. After 3 h, most Cx43 was modified by polyubiquitination and/or multiple monoubiquitination. Analysis of TRIM21/Cx43 complexes by high-performance size exclusion chromatography showed that complexes that consisted of highly phosphorylated Cx43 also contained ubiquitinated Cx43. Moreover, inhibition of the trafficking of Cx43 from the endoplasmic reticulum to the Golgi apparatus with Brefeldin A resulted in the near complete loss of both phosphorylated and ubiquitinated forms of Cx43. Mathematical modeling demonstrated that EGF-induced phosphorylation of Cx43 was associated with increased Cx43 ubiquitination. Collectively, these observations support the notion that Cx43 degradation is controlled by crosstalk between Cx43 phosphorylation and ubiquitination.

In addition to the above-mentioned E3 ubiquitin ligases, another member of the NEDD4 family, WWP1, has been shown to regulate Cx43 gap junctions in cardiomyocytes [124] and the E3 ubiquitin ligases LNX1 and -2 (ligand of NUMB Protein-X1 and -2) were recently found to have important roles in controlling the level of Cx36 gap junctions in the central nervous system [125], as is further discussed below.

## Regulation of Cx43 gap junctions by deubiquitinating enzymes

Cx43 ubiquitination is a reversible process, and two deubiquitinating enzymes, AMSH [associated molecule with the SH3 domain of STAM (signal transducing adaptor molecule)] and USP8 (ubiquitin carboxyl-terminal hydrolase 8) have been found to regulate Cx43 deubiquitination and lysosomal degradation (Fig. 3; Table 2) [107, 126].

### AMSH

AMSH has been shown to bind to Cx43, as determined by co-immunoprecipitation, and to partly co-localize with Cx43 at the plasma membrane and in intracellular compartments [126]. Ectopic overexpression of a catalytically inactive version of AMSH results in strongly increased levels of Cx43 modified by K63-linked polyubiquitin chains, which is associated with an increased Cx43 degradation rate. Upon overexpression of the catalytically inactive version of AMSH, Cx43 is observed on the limiting membrane of vesicular structures of the endolysosomal system, whereas the level of Cx43 at the plasma membrane is decreased [126]. A similar loss of Cx43 at the plasma membrane and an increase in Cx43 degradation are observed in response to AMSH depletion [126]. Accordingly, on overexpression of wild-type AMSH, the level of Cx43 at the plasma membrane is increased. Collectively, these data indicate that AMSH-mediated deubiquitination of Cx43 counteracts its endocytosis and lysosomal degradation, causing increased gap junction levels.

### USP8

USP8 was recently shown to bind to the C-terminal tail of Cx43, as determined by co-immunoprecipitation [107]. Ectopic overexpression of USP8 was found to promote the loss of both Cx43 monoubiquitination and polyubiquitin chains linked via Lys48 or Lys63, which was associated with increased Cx43 protein levels. In accordance with this finding, depletion of endogenous USP8 was associated with increased Cx43 ubiquitination and reduced Cx43 protein levels due to increased degradation via the autophagosomal pathway. This was correlated with loss of gap junctional intercellular communication [107].

## Emerging physiological and pathophysiological implications of connexin ubiquitination

Given the diverse physiological roles of gap junctions across different tissue types, connexin ubiquitination is expected to have a wide variety of important physiological implications. Below, we highlight recent studies that have started to elucidate the role of connexin ubiquitination in the regulation of gap junctions in the heart, the lens and the central nervous system and the implications of these findings for disease pathogenesis.

### Role of connexin ubiquitination in the regulation of gap junctions in the heart

The normal heart rhythm depends fundamentally on the coupling of cardiac myocytes by gap junctions [127]. The gap junctions are located at the intercalated discs, where they mediate the intercellular electrical coupling responsible for synchronous contraction of the heart [127]. The principal connexins expressed in cardiac myocytes are Cx43, Cx40 and Cx45, of which Cx43 is the most predominant [127]. Cx43 in the heart has a high turnover rate, and regulation of Cx43 degradation may be an important mechanism for modulating gap junctional communication in the heart under normal and pathophysiological conditions [128, 129]. Cx43 is highly remodeled in the diseased heart [127]. During ischemia, Cx43 undergoes rapid dephosphorylation, which is associated with electrical uncoupling and alteration in the distribution of Cx43 to the sides of the myocyte and to intracellular pools [130, 131].

Nielsen and colleagues have shown that Cx43 in neonatal ventricular rat cardiomyocytes is subjected to ubiquitination and binds to NEDD4 [132]. Moreover, stimulation of α-adrenergic Gα_q_-coupled receptors with noreprinephrine was found to cause increased Cx43 ubiquitination, which was suggested to be associated with increased internalization of gap junctions. Studies from Matesic’s group have identified a role for the E3 ubiquitin ligase WWP1, a member of the NEDD4 family, in regulating Cx43 degradation and gap junction size in cardiomyocytes. The authors demonstrated that global- or cardiomyocyte-specific overexpression of WWP1 in mice results in lethal ventricular arrhythmias around 8 weeks of age because of a reduction in Cx43 protein levels in the heart muscle [124]. Overexpression of WWP1 did not affect the Cx43 mRNA levels, suggesting that the WWP1-induced loss of Cx43 occurs at the post-translational level. In line with this notion, WWP1 was found to partly co-localize with Cx43 in small intracellular vesicular compartments of cardiomyocytes. Since WWP1 did not co-localize with Cx43 at the plasma membrane, it was suggested that WWP1 may not regulate gap junction internalization, but instead promote the degradation of Cx43 after internalization. Notably, overexpression of WWP1 in cardiomyocytes did not affect the protein levels of Cx40 and Cx45, suggesting that WWP1 specifically acts on Cx43. Furthermore, WWP1 overexpression had no effects on N-cadherin or desmoplakin protein levels, further corroborating the specific effect of WWP1 on Cx43. As determined by co-immunoprecipitation, Cx43 and WWP1 were found to bind to each other when exogenously expressed in human embryonic kidney 293T cells [124]. The binding was suggested to occur via the PY motif of Cx43. In line with the notion that WWP1 promotes Cx43 degradation, the Cx43 protein level in 293T cells was reduced in response to co-expression of WWP1, whereas a catalytically inactive version of WWP1 did not affect the Cx43 protein level. Moreover, the WWP1-induced loss of Cx43 protein levels was associated with a robust increase in Cx43 ubiquitination, whereas the inactive version of WWP1 did not affect the Cx43 ubiquitination status [124].

Studies from Girão’s group indicate that Cx43 ubiquitination is involved in the remodeling of gap junctions during acute cardiac ischemia. Using the atrial cardiomyocyte cell line HL-1 and organotypic heart cultures as model systems, they found ischemia to be associated with autophagy-mediated degradation of Cx43 [133]. The ischemia-induced degradation of Cx43 was shown to correlate with increased Cx43 ubiquitination and binding to Eps15 and p62, and depletion of p62 partly counteracted the degradation of Cx43 under these conditions. Increased ubiquitination and autophagy-mediated degradation of Cx43 in response to ischemia were also observed in organotypic heart cultures [133]. Moreover, ischemia was associated with enhanced Cx43 ubiquitination, as determined by the rat heart Langendorff perfusion model, and under these conditions, Cx43 was found to be modified by K63-linked ubiquitin chains [106]. Notably, counteracting Cx43 degradation during ischemia with chemical inhibitors of autophagy also prevents the loss of ischemia-associated gap junction intercellular communication [133]. This finding raises the possibility that targeting autophagy-mediated degradation of Cx43 could represent a potential therapeutic approach for preventing the loss of functional gap junctions during heart ischemia [133, 134]. The increase in Cx43 ubiquitination in response to ischemia is accompanied by recruitment of NEDD4 to intercalated discs, where it forms a complex with Cx43 [106]. However, depletion of NEDD4 does not counteract Cx43 ubiquitination or degradation induced by ischemia, suggesting that another E3 ubiquitin ligase may be involved in this process [133].

Shaw and colleagues have provided further insights into the crosstalk between Cx43 phosphorylation and ubiquitination and its implications for the loss of gap junctions during acute ischemia [67]. Exposing Langendorff-perfused mouse hearts to global ischemia was shown to result in phosphorylation of Ser368, in accordance with previous studies suggesting that phosphorylation of Cx43 at Ser368 is involved in gap junction remodeling during ischemia [135–137]. When a Cx43 construct in which Ser373 was mutated to alanine (Cx43-S373A) was transiently transfected into HaCaT human keratinocytes, it was found to have lost phosphorylation of Ser368, as well as Ser255, and to display more Cx43 at the plasma membrane than Cx43-wt did. Compared with Cx43-wt, Cx43-S373A also displayed strongly reduced ubiquitination, both under basal conditions and in response to TPA treatment. In accordance with these observations, TPA was found to induce loss of Cx43-wt at the plasma membrane because of increased internalization, whereas it did not affect the level of Cx43-S373A [67]. Cx43-S373A also displayed loss of binding to 14-3-3τ, whose normal function is to promote Cx43 internalization [67]. Collectively, these data indicate that phosphorylation of Cx43 at Ser373 promotes both Cx43 ubiquitination and binding to 14-3-3τ, and both events may contribute to reduced gap junction levels by promoting increased Cx43 internalization.

Gemel et al. have shown that some Cx40 mutants linked to atrial fibrillation have reduced stability as compared to Cx40-wt, when expressed in HeLa cells or HL-1 cardiomyocytes [98]. Among the mutants studied, Cx40-G38D showed the most dramatic differences from Cx40-wt and was, therefore, characterized in further detail. Both Cx40-wt and Cx40-G38D were demonstrated to be subjected to polyubiquitination and this modification was more prominent for the mutant. Moreover, proteasomal inhibition caused strongly increased levels of Cx40-G38D, which was associated with restoration of functional gap junctions. Collectively, these observations suggest that mutations in Cx40 may lead to atrial fibrillation by causing increased polyubiquitination and ERAD of the mutant protein [98]. The findings also raise the possibility that restoring the function of certain Cx40 mutants linked to atrial fibrillation by reducing their proteasomal degradation may have therapeutical implications [98].

Recently, Green and colleagues demonstrated that Cx43 ubiquitination is involved in modulating gap junction levels in cardiac cells in response to loss of desmoplakin, a key component of desmosomes [138]. Desmosomes are cadherin-based intercellular adhesive junctions that have important roles in maintaining the integrity of the myocardium by tethering the intermediate filament cytoskeleton to sites of cell–cell adhesion [139]. Desmoplakin physically links the intermediate filament to the desmosomal cadherin complex and also regulates various intracellular signaling pathways [139]. Mutations in desmoplakin can cause cardiac disease, including deadly arrhythmias [139]. In cardiac cells, desmosomes are structurally and functionally associated with both adherens junctions and gap junctions. Conditional knock-out of desmoplakin in mouse cardiac cells results in a significant reduction in Cx43 protein levels [140, 141]. Mechanistically, loss of desmoplakin causes activation of the ERK1/2-MAPK pathway, which then stimulates phosphorylation of Ser279 and Ser282 within the C-terminal tail of Cx43 [138]. In neonatal rat ventricular cardiomyocytes, this phosphorylation event was found to be associated with increased Cx43 ubiquitination and degradation in lysosomes [138].

### Role of connexin ubiquitination in the regulation of gap junctions in the lens

The lens is an avascular and transparent organ, whose cells are coupled by an extensive network of gap junctions that facilitates the exchange of ions and metabolites throughout the organ [142, 143]. The mature lens is composed of fiber cells covered with a single layer of epithelial cells on the anterior hemisphere. The lens continues to grow throughout life as epithelial cells at the lens equator differentiate into fiber cells. As new fiber cells arise, older cells are pushed toward the center and become mature lens fibers. The differentiation of epithelial cells into fiber cells involves cell elongation and loss of nuclei and organelles [142, 143].

The lens expresses three connexin isoforms in mammals: Cx43, Cx46 and Cx50 [142, 143]. These connexins display differential spatial distributions, which are related to their specific functions in different regions of the lens. Cx43 is predominantly expressed in epithelial cells, Cx46 is mainly expressed in fiber cells and Cx50 is expressed in both epithelial and fiber cells. Studies on mice with targeted deletion of the genes encoding Cx46 or Cx50 have demonstrated that Cx46 is essential for lens transparency, and Cx50 is important for lens growth and transparency [144, 145]. Mutations of the genes encoding Cx46 and Cx50 are one of the common causes of hereditary cataracts in humans [142, 143].

Takemoto and colleagues have demonstrated that Cx43 and Cx46 are expressed and regulated in a reciprocal manner in lens epithelial cells in a process that involves Cx43 ubiquitination [146]. Ectopic overexpression of Cx46 in NN1003A rabbit lens epithelial cells was found to cause reduced Cx43 protein levels due to proteasome-dependent degradation, which was associated with an increase in Cx43 ubiquitination. The level of Cx50 was not altered under these conditions, indicating that the effect was specific for Cx43. The study further demonstrated that the C-terminal tail of Cx46 was essential and sufficient to induce Cx43 degradation. Although the exact mechanisms involved in the Cx46-induced degradation of Cx43 remain to be determined, it was suggested that it could be due to increased ERAD [146].

Studies by Liu et al. have identified a novel connection between the ubiquitin–proteasome system, gap junctions and lens clarity [147]. Degradation of proteins by the ubiquitin–proteasome system in the lens was found to be perturbed by overexpression of a ubiquitin with a mutation in the Lys6 residue (ubiquitin-K6W). Although this mutant form of ubiquitin did not affect the ability of ubiquitin to form polyubiquitin chains, the ubiquitin conjugates formed under these conditions were not recognized by the proteasome and were thus resistant to proteasomal degradation [148]. Therefore, ubiquitin-K6W acts as a specific upstream dominant negative inhibitor of the ubiquitin–proteasome system. Expression of ubiquitin-K6W in lenses in vivo was shown to result in accumulation of ubiquitin conjugates, stabilization of numerous regulatory proteins and cell cycle arrest [149, 150]. It also slowed the differentiation of epithelial cells into fibers and the removal of nuclei [151]. Lenses expressing ubiquitin-K6W were found to have a significant increase in Ca^2+^ concentration [147]. The elevated Ca^2+^ concentration resulted in hyperactivation of calpain, one of the dominant proteases in mammalian lenses, and as a consequence increased cleavage of calpain substrates, causing developmental defects and cataracts. Interestingly, lenses expressing ubiquitin-K6W were found to have significantly higher levels of Cx43 protein, whereas the levels of Cx46 and Cx50 were decreased. As the mRNA levels for Cx43 were comparable in lenses expressing ubiquitin-K6W and ubiquitin-wt, it was suggested that the increased level of Cx43 in lenses expressing ubiquitin-K6W was due to impaired degradation rather than increased synthesis of Cx43. In accordance with this notion, lenses expressing ubiquitin-K6W were found to have accumulated ubiquitinated Cx43, much of which would be expected to have incorporated ubiquitin-K6W and, therefore, be resistant to degradation. The study further suggested that the increase in Ca^2+^ concentration in the lenses expressing ubiquitin-K6W was partly due to the increased Cx43 protein levels. Corroborating the in vivo data, ectopic expression of ubiquitin-K6W in human lens epithelial cells resulted in increased levels of ubiquitinated Cx43, which was associated with increased protein levels. Together, these observations suggest that Cx43 ubiquitination may have important roles in modulating Ca^2+^ concentration and, as a consequence, calpain activity in the lens. The findings also raise the possibility that exploiting the ubiquitin–proteasome system and the Ca^2+^-calpain pathway may provide new approaches to preventing or curing human cataracts [147].

In addition to Cx43, Cx45.6, the chick ortholog of mammalian Cx50, has been found to be modified by polyubiquitin chains when overexpressed in neuro2A (N2A) mouse neuroblastoma cells [109]. In accordance with this finding, the degradation of Cx45.6 in lens primary cultures was shown to be mediated primarily through the proteasomal pathway. Minogue et al. have shown that a mutant version of Cx50 that has a frameshift after amino acid 255 and causes recessive congenital cataracts undergoes strongly enhanced ERAD as compared to Cx50-wt when stably expressed in HeLa cells [152]. Accordingly, the cellular levels of the mutant Cx50 are considerably lower than that of Cx50-wt, and it rarely forms gap junctions. In line with the notion that the mutant Cx50 is subjected to ERAD, it is able to form functional gap junction plaques in response to proteasomal inhibition, which is associated with an increase in gap junction intercellular communication [152]. Moreover, the chemical inhibitor eeyarestatin I, which inhibits ERAD by targeting p97-associated deubiquitination [153], causes a considerably stronger increase in the cellular level of the mutant Cx50 than that of Cx50-wt. Under these conditions, both the mutant Cx50 and Cx50-wt are detected at higher apparent molecular masses in immunoblots, likely due to polyubiquitination [152].

### Role of connexin ubiquitination in the regulation of gap junctions in the central nervous system

Cx36 is, to date, the most abundantly detected connexin in neurons in mammals, and it forms the majority of electrical synapses in the mammalian central nervous system [154]. Cx36 is expressed in multiple types of neurons in the retina, brain and spinal cord and is critical in establishing synaptic circuitry during development and in providing for synchronous neuronal activity in the adult brain [154–157]. Cx36 null mice display functional deficits in various neural systems, including visual, motor and memory impairments. Electrical synapses are highly dynamic, and, similar to other connexins, Cx36 has a half-life of 1–3 h. Cx36 contains a PDZ [PSD95, DLGA, zonula occludens-1 (ZO-1)] domain interaction motif in its carboxy terminus through which it binds to the PDZ domain-containing proteins such as ZO1 [158].

LNX1 and -2 are RING E3 ubiquitin ligases that have multiple roles in the central nervous system. In addition to the RING domain, LNX1 and -2 contain four PDZ domains through which they interact with their substrate proteins [159, 160]. Recently, Nagy and colleagues identified LNX1 and -2 as important regulators of Cx36 gap junctions in neurons [125]. LNX1 and LNX2 were found to partly co-localize with neuronal gap junctions formed by Cx36 in rodent brain, as well as in N2A cells transfected with Cx36-enhanced cyan fluorescence protein (Cx36-eCFP). In accordance with this finding, as determined by co-immunoprecipitation, LNX1 was found to form a complex with Cx36 in mouse brain. Through pull-down assays, LNX1 and LNX2 were found to directly interact with Cx36 via its carboxy terminus. Co-transfection of N2A cells with Cx36-eCFP and LNX1 caused a strong loss of Cx36-eCFP-containing gap junctions, whereas co-transfection with Cx36-eCFP and an LNX1 mutant that lacked E3 ubiquitin ligase activity did not affect Cx36-eCFP gap junction levels [125]. A similar loss of gap junctions was observed in response to overexpression of LNX2, whereas a catalytically inactive form of LNX2 did not affect gap junction levels. Moreover, LNX2-wt, but not a catalytically inactive version, was found to increase Cx36-eCFP ubiquitination. The LNX2-induced loss of gap junctions was associated with reduced Cx36-eCFP protein levels due to increased lysosomal degradation. Collectively, these data add Cx36 to the list of substrate proteins of LNX1 and -2 in neurons and suggest that ubiquitination has an important role in controlling the plasticity of electrical synapses formed by Cx36-containing neuronal gap junctions.

## Conclusions and future perspectives

An increasing body of experimental data points toward a central role of connexin ubiquitination in the regulation of gap junction intercellular communication. The emerging picture is that connexins can become ubiquitinated at different subcellular localizations, such as the endoplasmic reticulum, the plasma membrane and endosomes, and that at each of these locations, connexin ubiquitination may play distinct roles. In accordance with this notion, connexins have been demonstrated to be modified with different types of ubiquitination. Each of these types of ubiquitin modifications of connexins may, in principle, be recognized by different effector proteins containing ubiquitin-binding domains that link connexin ubiquitination to a specific downstream event, such as proteasomal degradation, endocytosis or lysosomal sorting of connexins. In recent years, researchers have also started to shed light on the physiological roles of connexin ubiquitination in specific tissue types and how dysregulation of these processes may contribute to loss of functional gap junctions during disease pathogenesis.

Among the key challenges ahead is to obtain a more comprehensive understanding of the molecular basis by which E3 ubiquitin ligases and deubiquitinating enzymes, as well as other components of the ubiquitin system, control gap junction intercellular communication. The continuous technological developments in the fields of mass spectrometry and proteomics are likely to set the stage for new advances in this research area. In the future, it will also be interesting to further investigate the cell- and tissue-type specific roles of E3 ubiquitin ligases and deubiquitinating enzymes in the regulation of gap junctions using relevant mouse knock-out and knock-in models. A possible scenario is that the components of the ubiquitin conjugation machinery involved in controlling connexin ubiquitination and gap junction levels vary considerably between different cell types as well as between connexin isoforms.

Dysregulation of protein ubiquitination is involved in numerous human diseases, including cancer, neurodegenerative diseases, and autoimmunity and inflammatory disorders [92]. Components of the ubiquitin system are, therefore, attractive drug targets [161]. So far, relatively few drugs targeting the ubiquitin system have been approved for clinical use [161]. However, new technical advances, combined with an increased knowledge about the ubiquitination process, are likely to spur progress in this area of drug development [92, 161]. As our understanding of the role of ubiquitination in the regulation of connexins increases, the development of new drugs that target the ubiquitin system could offer novel therapeutic opportunities for restoring functional gap junctions in human disease states.
